# Effect of Structured Nutrition Education on Maternal and Infant and Young Child Feeding (IYCF)-Related Knowledge and Child Growth: A Randomized Controlled Trial in Urban Slums of Belagavi, India

**DOI:** 10.7759/cureus.107682

**Published:** 2026-04-24

**Authors:** Anagha P Kappadan, Mubashir Angolkar, Melkey S Bunyan, Nicolas Naorem

**Affiliations:** 1 Department of Radiation Oncology, Christian Medical College, Ludhiana, IND; 2 Department of Public Health, KLE Academy of Higher Education and Research, Jawaharlal Nehru Medical College (JNMC), Belgaum, IND; 3 Department of Neurology, Christian Medical College, Ludhiana, IND

**Keywords:** child nutrition, infant and young child feeding practices, mothers knowledge attitude and practice, nutrition education, public health

## Abstract

Background: Child malnutrition remains a critical public health crisis in India, particularly within urban slum populations where suboptimal infant and young child feeding (IYCF) practices are prevalent. Maternal knowledge and behavior serve as the primary determinants of dietary intake and physical growth during the first two years of life. This study evaluated the effectiveness of a structured nutrition education intervention delivered to mothers of children aged six to 24 months attending Anganwadi centers in Belagavi, India.

Methods: A randomized controlled trial was conducted involving 62 mother-child pairs recruited from urban slum Anganwadi centers. Participants were randomized into an intervention group of 31 (50%) participants and a control group of 31 (50%) participants. The intervention group received structured nutrition education via PowerPoint (Redmond, WA: Microsoft Corp.) presentations and handouts (pamphlets) covering breastfeeding, complementary feeding, and dietary diversity, while the control group received only standard pamphlets. Maternal knowledge, attitudes, and practices (KAP) and child anthropometric measurements (weight, height, mid-upper arm circumference {MUAC}) were assessed at baseline and at a three-month follow-up.

Results: At baseline, both groups were comparable across sociodemographic and nutritional indicators. Post-intervention, the intervention group demonstrated significantly higher knowledge scores (16.29±3.34) compared to the control group (14.16±2.40; p<0.01). Specifically, maternal awareness of early breastfeeding initiation in the intervention group increased from 15 (48.4%) to 28 (90.3%), and knowledge of exclusive breastfeeding rose from five (16.1%) to 24 (77.4%). Practice scores were significantly higher in the intervention group (19.13±1.63) than in the control group (17.68±1.33; p<0.01). Reported practices of early initiation and exclusive breastfeeding in the intervention group improved from 19 (62.5%) to 31 (100%). Although maternal knowledge and practices improved significantly, no significant post-intervention difference in attitude scores was observed between groups. While weight-based indicators showed no significant differences, height-for-age Z-score (HAZ) remained significantly better in the intervention group (p<0.01) compared to the control group.

Conclusions: A structured, community-based nutrition education intervention significantly enhances maternal IYCF knowledge and practices. These behavioral improvements were associated with stabilized HAZ scores in the intervention group, suggesting that targeted maternal education is a feasible and effective strategy to address malnutrition and support growth stabilization in underprivileged urban settings.

## Introduction

Malnutrition is a major public health challenge in India. According to the World Health Organization (WHO), malnutrition refers to deficiencies or excesses in nutrient intake, an imbalance of essential nutrients, or impaired nutrient utilization. The burden of malnutrition consists of both undernutrition and overweight/obesity, as well as diet-related non-communicable diseases. Undernutrition manifests in the following four broad forms: wasting, stunting, underweight, and micronutrient deficiencies [1].

Child malnutrition is a global health problem and a major risk factor for child death [2]. It was estimated that 149 million children under the age of five years were stunted, 45 million wasted, and 37 million were overweight or obese worldwide in 2022 [3]. The National Family Health Survey 5 (NFHS-5) in India reported that in 2019-2020, 32.1% of children under five years were underweight, 19.3% were wasting, and 35.5% were stunted. In Karnataka in 2019-2020, 32.2% of children under five years were found to be stunted, 18.5% were wasting, and 29.4% were underweight, according to the NFHS-5 [4]. Around 36.9% of the population in Belgaum, Karnataka, is underweight, 32.8% is stunted, and 23.6% is wasting [5].

Childhood malnutrition manifests as stunting (chronic deprivation), which permanently impairs physical and intellectual development, or wasting (acute deprivation), which weakens the immune system and increases mortality risk. Children who are underweight face a significantly higher risk of death, even in moderate cases. Meanwhile, childhood obesity often persists into adulthood, leading to chronic conditions like diabetes and cardiovascular disease [6]. Sustainable Development Goals (SDG) target 2.2 - end all types of malnutrition, including meeting the globally agreed-upon targets for stunting and wasting among children under five years by 2025. It also addresses the nutritional requirements of older adults, pregnant and lactating women, and teenage girls [7]. Infants and children are more vulnerable members of the population because they require more nutrition than adults do in order to grow and develop [8]. It may be caused by inadequate nursing techniques, low-quality complementary foods, harmful feeding methods, and tainted complementary foods and equipment [9].

As a crucial component of initiatives to combat child malnutrition and mortality, the WHO encourages the adoption of the infant and young child feeding (IYCF) strategy for children aged six to 24 months [10]. Feeding infants and young children is essential for promoting the survival of children and healthy development and growth [11]. A child's first two years of life offer a crucial window of time for ensuring survival, growth, and development through the use of the best IYCF methods. The rate of child mortality can be decreased by adhering to optimal breastfeeding practices, which include starting the breastfeeding process within 1 h of the baby's birth, exclusive breastfeeding (EBF) for six months, continuing breastfeeding for up to two years after the baby is born, and providing age-appropriate supplemental feeding [12]. Therefore, it is imperative to enhance IYCF practices for children 0-24 months of age in order to promote better nutrition, health, and development [13].

Maternal nutrition knowledge is a critical determinant in preventing childhood malnutrition and stunting, as it empowers caregivers to correct suboptimal feeding habits and address chronic nutritional imbalances that lead to developmental failure [14]. Enhancing a mother’s understanding of balanced diets remains one of the most effective strategies for reducing the prevalence of malnutrition, given the direct influence of maternal behavior on early childhood growth outcomes [2]. In this study, Anganwadi centers were selected as the primary delivery platform. This choice was informed by their established infrastructure within the Integrated Child Development Services (ICDS) scheme and their capacity to reach vulnerable populations through trusted community health workers. Despite the high burden of malnutrition in urban Karnataka, there remains a paucity of implementation research evaluating structured educational interventions within the specific sociocultural context of urban slum Anganwadi networks. The primary objective of this study was to evaluate the effectiveness of a structured nutrition education intervention on maternal knowledge, attitudes, and practices (KAP) regarding IYCF. The secondary objective was to assess the intervention's impact on the anthropometric status and growth stabilization of children aged six to 24 months attending Anganwadi centers in Belagavi, India.

## Materials and methods

Study design

A community-based randomized controlled trial (RCT) was conducted across selected Anganwadi centers in the Belagavi district of Karnataka, India. The study specifically targeted urban slum populations in the Ram Nagar and Kacheri Galli areas, spanning a one-year period from March 2023 to March 2024. Participants consisted of mother-child dyads, specifically including mothers and their children aged six to 24 months, recruited through a network of local Anganwadi centers.

Eligibility criteria

To ensure the study’s internal validity, participants were selected based on specific inclusion and exclusion criteria. Mothers were eligible for inclusion if they were between 20 and 40 years of age, had a child aged six to 24 months, and were permanent residents of the designated study area. Conversely, the study excluded mothers of children with major comorbidities, such as cancer, neurological illnesses, or congenital heart disease, as well as any mothers who did not provide informed consent to participate.

Ethical considerations

Ethical approval was obtained from the Institutional Ethics Committee, Jawaharlal Nehru Medical College, Belagavi. Administrative permission was secured from the Child Development Project Officer (CDPO), Belagavi. The trial was prospectively registered in the Clinical Trials Registry - India (#CTRI/2024/02/063016).

Sample size

To ensure clinical relevance, the study was powered using mid-upper arm circumference (MUAC), a validated WHO-standardized screening tool for acute malnutrition in children aged six to 23 months, as it represents a key secondary objective requiring high statistical precision. Using a single mean paired t-test formula, the sample size was powered to detect a clinically significant improvement of 0.5 cm in mean MUAC (projected from a baseline of 12.3±0.5 to 12.8±0.6 cm post-intervention). This difference corresponds to a large effect size (Cohen’s d=0.734). With a statistical power of 90%, a two-sided alpha error of 1%, and a 95% confidence level, the minimum required sample size was estimated at 31 participants per group. A total of 62 mother-child pairs were enrolled (31 in the intervention group and 31 in the control group). By powering the study based on the more conservative anthropometric variable (MUAC), the study ensured more than sufficient statistical power to detect significant changes in the primary outcome of maternal KAP scores.

Randomization and intervention allocation

**Figure 1:**
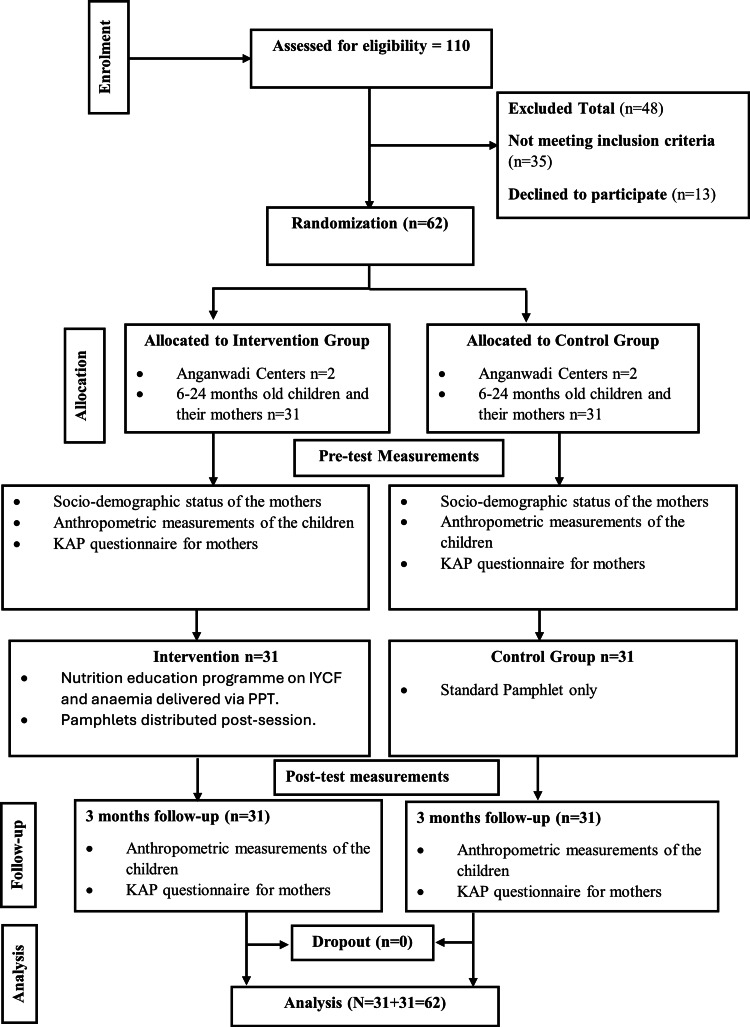
Consolidated Standards of Reporting Trials (CONSORT) flow chart. KAP: knowledge, attitudes, and practices

Four Anganwadi centers were purposively identified from the urban slum areas of Ram Nagar and Kacheri Galli to ensure geographic separation. These centers were then randomly allocated to either the intervention (n=2 centers) or control (n=2 centers) arm using simple random allocation. To ensure baseline comparability between the clusters, sociodemographic variables (maternal age, education, and family income) were analyzed across the four centers prior to allocation; no significant inter-center differences were observed (p>0.05). To minimize the risk of contamination, cluster-level allocation was employed. Since all mothers within a specific Anganwadi center received the same protocol (either intervention or control), the potential for informal exposure to intervention content between groups was significantly reduced. Furthermore, participants were requested to refrain from sharing educational pamphlets with individuals outside their immediate center during the study period. A total of 110 mother-child pairs were initially screened for eligibility. Of these, 48 pairs were excluded (35 did not meet the inclusion criteria, and 13 declined to participate), leaving a final sample of 62 participants. Following baseline assessments, 31 dyads from the intervention centers and 31 from the control centers were enrolled. To ensure objectivity, while blinding of participants and the primary investigator was not feasible due to the educational nature of the study, the data analyst remained blinded to group assignments. The trial flow followed the Consolidated Standards of Reporting Trials (CONSORT) 2010 guidelines (Figure 1) [15].

Study instrument

This study used a structured knowledge, attitude, and practice (KAP) questionnaire specifically developed to assess infant and young child feeding (IYCF) behaviors (table in appendix). The instrument comprised 43 items across the following three domains: knowledge (17 items) on breastfeeding and complementary feeding; attitude (12 items) using a three-point Likert scale (0=disagree, 1=neutral, 2=agree); and practice (14 items) documenting feeding behaviors and the eight WHO food groups. Knowledge items were scored as binary (correct=1, incorrect=0; max: 17), while the attitude and practice domains had maximum scores of 24 and 22, respectively. The study tool underwent a rigorous content validation process. A panel of five experts in pediatrics and public health evaluated each item for relevance, clarity, and simplicity on a four-point scale. The Item-level Content Validity Index (I-CVI) ranged from 0.80 to 1.00, and the Scale-level Content Validity Index (S-CVI/Ave) was calculated at 0.86, indicating high content validity. Internal consistency was further confirmed via a pilot study with 10 non-study pairs, yielding a Cronbach’s alpha of 0.81, indicating high reliability and instrument stability.

Intervention and follow-up procedures

The intervention was delivered through organized group meetings held at the local Anganwadi centers. At the outset, a baseline assessment was conducted to collect sociodemographic data and administer a structured KAP questionnaire to evaluate maternal KAP of IYCF. All anthropometric measurements, including weight, height, MUAC, head circumference (HC), and chest circumference (CC), were performed following standardized World Health Organization (WHO) protocols. To ensure data quality, all measurements were taken by the primary investigator and a trained assistant. Inter-observer reliability was assessed prior to the study via a pilot session with 10 non-study pairs, yielding a technical error of measurement (TEM) within the acceptable range (>0.90 correlation).

**Figure 2:**
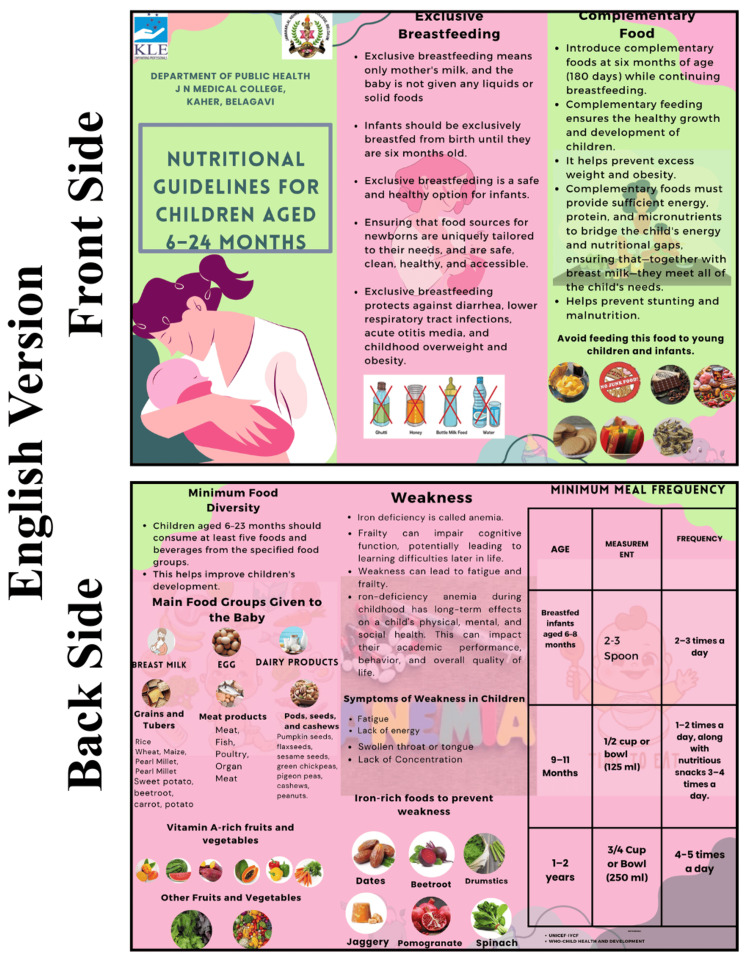
Institutional Ethics Committee (IEC) material English version.

**Figure 3:**
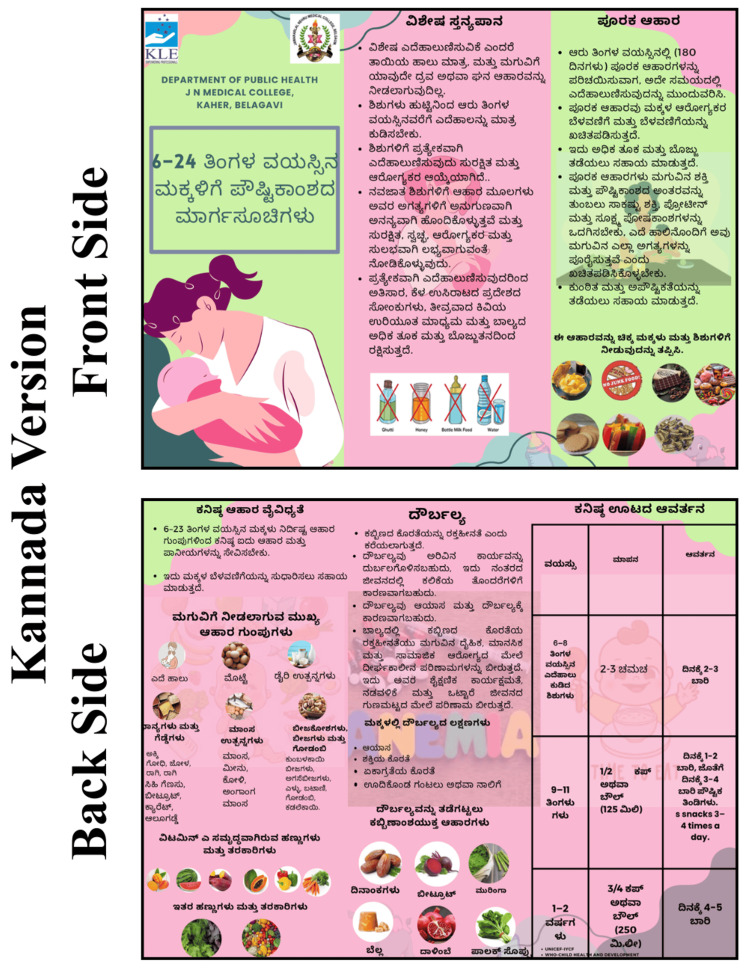
Institutional Ethics Committee (IEC) material Kannada version.

**Figure 4:**
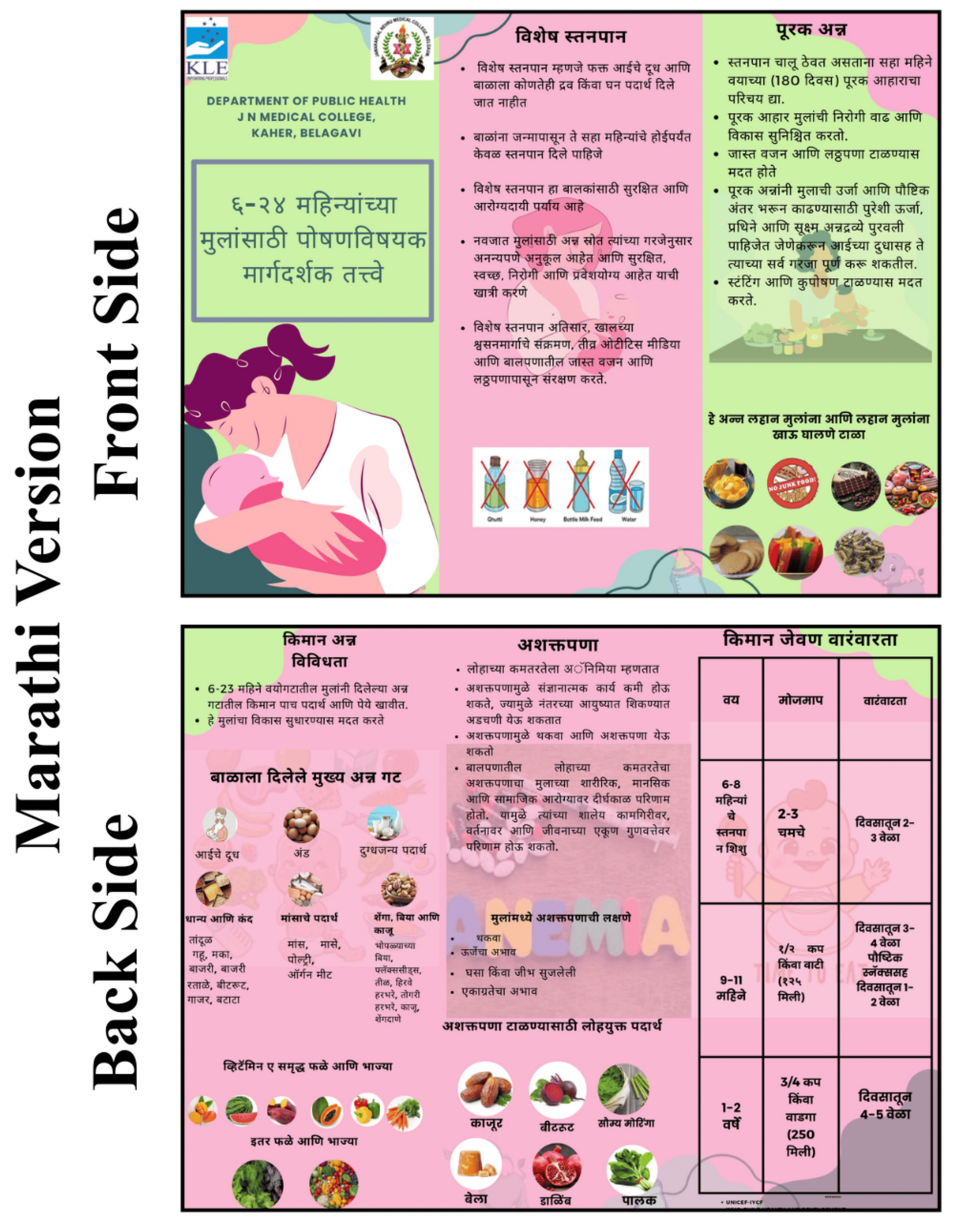
Institutional Ethics Committee (IEC) material Marathi version.

The intervention group participated in structured nutrition education sessions utilizing PowerPoint-based lectures. This curriculum emphasized critical IYCF components as follows: early initiation of breastfeeding, exclusive breastfeeding, appropriate complementary feeding, minimum dietary diversity, meal frequency, and anemia prevention. Additionally, mothers were educated on identifying signs of malnutrition, interpreting growth charts, and managing common childhood ailments, such as diarrhea and fever, at home. These educational efforts were reinforced through the distribution of a nutritional module and structured pamphlets (Figures 2-4). These resources outlined the "golden rules" of feeding, including colostrum feeding and exclusive breastfeeding for 180 days, and provided guidance on incorporating iron-rich foods like spinach, beetroot, dates, and jaggery. Specific feeding schedules were also provided, detailing age-appropriate meal frequencies and portion sizes for children aged six to eight, nine to 11, and 12 to 24 months. In contrast, the control group received only the standard educational pamphlets without the lecture-based sessions. These materials were adapted from the official IYCF guidelines provided by the Ministry of Health and Family Welfare (MoHFW), Government of India, and the Integrated Child Development Services (ICDS) curriculum.

The study employed a monitoring approach over a three-month follow-up period. Monthly reinforcement meetings were conducted at the Anganwadi centers to address maternal queries and solidify the core messages of the intervention. Final post-intervention assessments of child growth parameters and maternal KAP scores were performed at the conclusion of the third month. The study achieved a 100% session completion rate among the 62 enrolled participants, as education was delivered during scheduled Anganwadi visits, resulting in no attrition post-randomization.

Data analysis

Data were entered and coded in Microsoft Excel (Redmond, WA: Microsoft Corp.) and analyzed using SPSS version 20.0 (Armonk, NY: IBM Corp.). Anthropometric indices were analyzed using WHO Anthro software (Geneva, Switzerland: WHO) to generate Z-scores and categorize nutritional status according to WHO growth standards [16]. The normality of data was assessed using the Shapiro-Wilk test. Descriptive statistics (mean, SD, and percentages) summarized sociodemographic data.

Baseline comparability between the intervention and control groups was assessed using the chi-square test for categorical variables and the Mann-Whitney U test for continuous variables. The analysis was conducted on an intention-to-treat (ITT) basis. To evaluate the effectiveness of the intervention, within-group changes (pre-intervention vs. post-intervention) were analyzed using the Wilcoxon signed-rank test, while between-group comparisons for non-normally distributed continuous variables (KAP scores and anthropometric Z-scores) were performed using the Mann-Whitney U test. A p<0.05 was considered statistically significant.

## Results

Sociodemographic profile

Analysis of the baseline sociodemographic profile revealed that the study included 62 mothers, with 31 participants each in the intervention and control groups (Table 1). Most mothers belonged to the 20-30 years age group, accounting for 25 (80.6%) in the intervention group and 23 (74.2%) in the control group, while six (19.4%) and eight (25.8%) mothers, respectively, were aged 31-40 years. The mean age of mothers was 26.64±3.9 years in the intervention group and 28.06±4.8 years in the control group. All participants were married (100%). High school education was the most common educational status, reported by 15 (48.4%) mothers in the intervention group and 11 (35.5%) mothers in the control group, followed by pre-university college (PUC)/diploma in eight (25.8% in both groups) mothers, and graduation by four (12.9%) mothers in the intervention group and seven (22.6%) in the control group. Post-graduation was reported by one (3.2%) mother in the intervention group and three (9.7%) mothers in the control group, while primary schooling was reported by three (9.7%) and one (3.2%) mother, respectively. Nearly all mothers were unemployed and housewives, 30 (96.8%) in both groups, with only one mother, one (3.2%) in each group, being employed as a private employee. Joint family structure was observed among 17 (54.8%) mothers in the intervention group and 15 (48.4%) in the control group, while nuclear families were observed among 14 (45.2%) and 16 (51.6%) mothers, respectively. Socioeconomic status assessment using the Modified B.G. Prasad classification showed that most mothers belonged to the lower middle-class category, comprising 18 (56.1%) in the intervention group and 12 (38.7%) in the control group.

**Table 1:** Sociodemographic characteristics of participating mothers in intervention and control groups. P<0.05 was statistically significant. PUC: pre-university college

Variables	Groups		Test statistic χ^2^	p-Value
Age of the mothers (years)	Intervention, n (%)	Control, n (%)		
20-30	25 (80.6)	23 (74.2)	0.379	0.53
31-40	6 (19.4)	8 (25.8)		
Marital status of mother				
Married	31 (100)	31 (100)	1.016	0.31
Divorced	0 (0)	0 (0)		
Widow	0 (0)	0 (0)		
Educational status of mothers				
Post-graduate	1 (3.2)	3 (9.7)	4.43	0.48
Graduate	4 (12.9)	7 (22.6)		
PUC or diploma	8 (25.8)	8 (25.8)		
High school	15 (48.4)	11 (35.5)		
Middle school	0 (0)	1 (3.2)		
Primary school	3 (9.7)	1 (3.2)		
Occupational status of mothers				
Employed	1 (3.2)	1 (3.2)	0.0	1.0
Unemployed	30 (96.8)	30 (96.8)		
Occupation of mothers				
Government employee	0 (0)	0 (0)	0.0	1.0
Private employee	1 (3.2)	1 (3.2)		
Self-employment or business	0 (0)	0 (0)		
Daily wage worker	0 (0)	0 (0)		
Housewife	30 (96.8)	30 (96.8)		
Others	0 (0)	0 (0)		
Family type				
Nuclear	14 (45.2)	16 (51.6)	1.159	0.56
Joint	17 (54.8)	15 (48.4)		
Family income				
Lower class	7 (22.6)	3 (9.7)	8.672	0.07
Lower middle class	18 (56.1)	12 (38.7)		
Middle class	3 (9.7)	12 (38.7)		
Upper middle class	2 (6.5)	2 (6.5)		
Upper class	1 (3.2)	2 (6.5)		
Total	31 (100)	31 (100)	-	-

In the intervention group, 16 (51.6%) participants were female, and 15 (48.4%) were male. In the control group, 16 (51.6%) were males, and 15 (48.4%) were females. Age-specific distribution showed that in the intervention group, 15 (48.4%) children were aged six to 12 months, and eight (25.8%) were in both the 12-18 and 18-24 months age groups. In the control group, 17 (54.8%) were aged six to 12 months, six (19.4%) were aged 12-18 months, and eight (25.8%) were aged 18-24 months (Table 2).

**Table 2:** Comparison of child demographic characteristics between study groups. P<0.05 was statistically significant.

Variables	Groups		Test statistic χ^2^	p-Value
Gender	Intervention, n (%)	Control, n (%)		
Male	15 (48.4)	16 (51.6)	0.065	0.79
Female	16 (51.6)	15 (48.4)		
Age of children in months				
6-12	15 (48.4)	17 (54.8)	0.411	0.81
12-18	8 (25.8)	6 (19.4)		
18-24	8 (25.8)	8 (25.8)		
Total	31 (100)	31 (100)	-	-

Baseline anthropometric measurements

Baseline anthropometric measurements of children showed similar growth and nutritional status in both groups at the start of the study (Table 3). In the intervention group, mean (±SD) height, weight, MUAC, chest circumference (CC), and head circumference (HC) were 75.55±6.28 cm, 9.39±1.87 kg, 14.03±1.22 cm, 45.26±2.50 cm, and 45.42±2.05 cm, respectively. Corresponding values in the control group were 72.32±5.60 cm, 8.87±1.38 kg, 13.65±0.92 cm, 44.23±2.32 cm, and 44.55±1.98 cm. Overall, these findings confirm that both groups were comparable at baseline, ensuring that any post-intervention differences can be more confidently attributed to the intervention.

**Table 3:** Baseline anthropometric characteristics of children in intervention and control groups. Min: minimum value; Max: maximum value; MUAC: mid-upper arm circumference; CC: chest circumference; HC: head circumference

Variables	Mean±SD	Median	IQR	Min	Max
Intervention group					
Height (cm)	75.55±6.276	75.00	9	62	86
Weight (kg)	9.39±1.874	10.00	2	6	14
MUAC (cm)	14.03±1.224	14.00	2	11	16
CC (cm)	45.26±2.503	45.00	3	40	50
HC (cm)	45.42±2.046	46.00	3	42	49
Control group					
Height (cm)	72.32±5.6	72.00	7	59	82
Weight (kg)	8.87±1.384	9.00	2	6	11
MUAC (cm)	13.65±0.915	14.00	1	12	16
CC (cm)	44.23±2.32	45.00	9	39	48
HC (cm)	44.55±1.977	44.00	2	39	48

Maternal knowledge, attitude, and practice (KAP) and child nutritional status (Z-scores)

At baseline, a significant group difference in height-for-age Z-score (HAZ) and attitude scores was observed (p<0.01); however, other sociodemographic and nutritional parameters remained comparable. Knowledge scores were similar between the groups at baseline (p=0.71), but post-intervention, the intervention group demonstrated significantly higher knowledge (16.29±3.34 vs. 14.16±2.40; p<0.01). While attitude scores were significantly higher in the control group at baseline (p<0.01), no significant difference was observed post-intervention (p=0.92), indicating a relative improvement and stabilization of attitudes within the intervention group. Practice scores did not differ at baseline (p=0.10), but post-intervention, the intervention group showed significantly higher practice levels (19.13±1.63 vs. 17.68±1.33; p<0.01).

Nutritional status outcomes revealed no significant differences in weight-for-height Z-score (WHZ) or weight-for-age Z-score (WAZ) at either baseline or post-intervention. Regarding linear growth, although HAZ scores were significantly higher in the intervention group at both baseline (p<0.01) and post-intervention (p<0.01), the control group exhibited a more pronounced decline over time. These findings indicate that the structured educational intervention effectively improved maternal knowledge and practices, stabilized attitudes, and positively influenced growth stabilization in children compared to the control group (Table 4).

**Table 4:** Impact of nutrition education intervention on maternal knowledge, attitude, practices, and anthropometric indices. *P<0.05 was statistically significant. WAZ: weight-for-age Z-score; HAZ: height-for-age Z-score; WHZ: weight-for-height Z-score

Tests	Groups	Mean	SD	Mann-Whitney U test	
				p-Value	U-value
Knowledge pre-test	Intervention	9.35	2.288	0.71	506
	Control	9.52	1.947		
Knowledge post-test	Intervention	16.29	3.339	<0.01*	285
	Control	14.16	2.396		
Attitude pre-test	Intervention	6.94	2.407	<0.01*	672.5
	Control	8.61	2.171		
Attitude post-test	Intervention	10.74	1.548	0.92	474.5
	Control	10.74	1.505		
Practice pre-test	Intervention	16.16	2.208	0.10	365.5
	Control	15.26	2.081		
Practice post-test	Intervention	19.13	1.628	<0.01*	246
	Control	17.68	1.326		
WHZ pre	Intervention	-0.19	1.493	0.25	558.5
	Control	0.19	1.108		
WHZ post	Intervention	0.19	1.470	0.07	606
	Control	0.84	1.186		
HAZ pre	Intervention	-0.48	1.503	<0.01*	239
	Control	-1.87	1.648		
HAZ post	Intervention	-1.48	1.411	<0.01*	278
	Control	-2.48	1.387		
WAZ pre	Intervention	-0.52	1.338	0.26	404
	Control	-0.87	1.231		
WAZ post	Intervention	-0.55	1.312	1.00	480.5
	Control	-0.55	1.091		

A within-group comparison of maternal KAP scores and child anthropometric indices was performed using the Wilcoxon signed-rank test to evaluate changes over the three-month study period. In both the intervention and control groups, maternal KAP scores demonstrated statistically significant improvements (p<0.01); however, the intervention group exhibited a more pronounced increase in knowledge (mean change of 6.94 vs. 4.64 in control), suggesting that structured nutrition education provided a superior gain in IYCF literacy compared to standard materials. Regarding anthropometric outcomes, both groups showed significant shifts in WHZ, with the intervention group’s mean moving from a negative to a positive value (p=0.02), indicating improved acute nutritional status and growth stabilization. Conversely, HAZ significantly declined in both arms (p=0.01), shifting from -0.48 to -1.48 in the intervention group and -1.87 to -2.48 in the control group. This trend suggests that while educational interventions can effectively stabilize weight and enhance maternal feeding practices in the short term, the underlying chronic challenge of linear growth faltering (stunting) persists in urban slum environments and may require more prolonged multi-sectoral support. Finally, the intervention group maintained stability in WAZ (p=0.83), whereas the control group showed a significant increase (p=0.02), likely reflecting a more balanced weight management profile in the intervention arm despite the observed lag in linear growth (Table 5).

**Table 5:** Within-group comparison of maternal KAP scores and child anthropometric indices pre- and post-intervention in both the groups. *P<0.05 was statistically significant. WAZ: weight-for-age Z-score; HAZ: height-for-age Z-score; WHZ: weight-for-height Z-score; KAP: knowledge, attitudes, and practices

Groups	Mean	SD	SE mean	Wilcoxon signed-rank test
				p-Value
Intervention group				
Knowledge pre	9.35	2.288	0.411	0.01*
Knowledge post	16.29	3.339	0.600	
Attitude pre	6.94	2.407	0.432	0.01*
Attitude post	10.74	1.548	0.278	
Practice pre	16.16	2.208	0.396	0.01*
Practice post	19.13	1.628	0.292	
WHZ pre	-0.19	1.493	0.268	0.02*
WHZ post	0.19	1.470	0.264	
HAZ pre	-0.48	1.503	0.270	0.01*
HAZ post	-1.48	1.411	0.253	
WAZ pre	-0.52	1.338	0.240	0.83
WAZ post	-0.55	1.312	0.236	
Control group				
Knowledge pre	9.52	1.947	0.350	0.01*
Knowledge post	14.16	2.396	0.430	
Attitude pre	8.61	2.171	0.390	0.01*
Attitude post	10.74	1.505	0.270	
Practice pre	15.26	2.081	0.374	0.01*
Practice post	17.68	1.326	0.238	
WHZ pre	0.19	1.108	0.199	0.01*
WHZ post	0.84	1.186	0.213	
HAZ pre	-1.87	1.648	0.296	0.01*
HAZ post	-2.48	1.387	0.249	
WAZ pre	-0.87	1.231	0.221	0.02*
WAZ post	-0.55	1.091	0.196	

## Discussion

The results of this study demonstrate that structured nutrition education can substantially improve maternal KAP regarding IYCF, with meaningful impacts on child growth stabilization. Analysis of the baseline profile revealed that the groups were comparable in most sociodemographic aspects, with nearly half of the mothers having completed high school (48.4% intervention vs. 35.5% control) and a vast majority being unemployed (96.8%), consistent with similar maternal health studies in India and Saudi Arabia [17-20,21-23].

Maternal knowledge improved markedly following the intervention. Specifically, awareness of early breastfeeding initiation increased from 48.4% to 90.3%, outperforming post-intervention rates reported in similar studies in India (60%) and Saudi Arabia (61.7%) [24-26]. These findings suggest that the structured, audiovisual delivery method used in this study may be more effective than traditional counseling in enhancing retention of critical infant feeding information.

Maternal attitudes and the ceiling effect

Maternal attitudes and practices also showed substantial improvement. Notably, a significant baseline imbalance was observed, with the control group having higher attitude scores than the intervention group (p<0.01). However, this gap was equalized post-intervention (p=0.92). This convergence suggests a "ceiling effect," where the structured intervention enabled the intervention group to reach the maximum threshold of the measurement scale, effectively neutralizing the initial disparity. While regression to the mean is a possibility in longitudinal assessments, the parallel 100% achievement in practice scores for early initiation and exclusive breastfeeding (EBF) reinforces that this was a genuine shift in maternal intent and behavior rather than a statistical artifact.

Anthropometric outcomes and follow-up duration

Regarding anthropometric outcomes, measurements showed relative stability. It is important to note that weight-for-age (WAZ) and weight-for-height (WHZ) Z-scores did not show significant between-group divergence at the three-month mark. This is directly attributable to the study’s short follow-up duration; three months represents a minimal physiological window for observing significant shifts in total body mass or the reversal of wasting in a community-based setting without supplemental therapeutic feeding. However, the maintenance of significantly higher HAZ scores in the intervention group (p=0.01) compared to the control group, which saw a more pronounced decline, suggests that the intervention may exert a protective effect against further linear growth faltering.

Contextual comparisons

When comparing these results to international literature, contextual differences in study design must be acknowledged. For instance, research in Ghana reported no significant anthropometric shifts over a six-month period, potentially due to different baseline nutritional loads and a less structured educational curriculum [27]. Conversely, studies in Ethiopia utilized longer intervention durations and larger clusters, which may account for more varied effect sizes in practice scores [28]. These variations highlight that while structured education through community platforms such as Anganwadi centers is effective, the magnitude of anthropometric impact depends heavily on local sociocultural factors and the duration of follow-up.

Study limitations

Several limitations warrant consideration in the interpretation of these findings. First, the sample size was relatively small and drawn from a restricted geographic area within a specific urban slum network, which may limit the generalizability of the results to broader or more diverse populations. Second, a significant baseline imbalance was observed in maternal attitude scores and child HAZ between the groups (p<0.01). While such disparities are a recognized risk of simple randomization in smaller cohorts, the use of within-group analyses and the subsequent post-intervention equalization of attitude scores (p=0.92) suggest a robust intervention effect despite these initial variances.

The study’s three-month intervention and follow-up period represents another constraint. While this duration was sufficient to measure significant shifts in maternal KAP and acute growth stabilization, it constitutes a minimal physiological window for observing meaningful reversals in WAZ or chronic linear growth faltering (HAZ). Furthermore, the educational materials provided to the control group were standard-of-care pamphlets issued by the presiding health authorities. Since the investigators did not influence the design or content of these standard materials, their independent impact on the control group's outcomes remains a potential confounding variable.

Finally, blinding of participants and the primary investigator was not feasible due to the nature of the educational delivery, which may have introduced reporting or performance bias. Despite these constraints, the study demonstrates the practical effectiveness of leveraging existing ICDS infrastructure to deliver structured, high-impact nutrition education to high-risk communities.

## Conclusions

This study demonstrates that structured nutrition education is a powerful tool for improving maternal KAP regarding IYCF. Mothers who received the intervention showed significant gains in critical areas, including timely initiation of breastfeeding, adherence to exclusive breastfeeding, and appropriate implementation of complementary feeding. These behavioral shifts were associated with a stabilization of HAZ scores in the intervention group, suggesting that targeted maternal education provides tangible health benefits for children in underprivileged settings.

The findings highlight the profound impact of engaging mothers with practical, evidence-based nutritional information. Even a focused, three-month intervention can empower caregivers to make informed decisions that positively influence growth and development. While limited by a relatively small sample size, a short follow-up period, and baseline variances, the results underscore the potential of nutrition education as a feasible and effective public health strategy. Integrating such structured programs into existing maternal and child health frameworks, such as Anganwadi services, could play a vital role in addressing the burden of childhood malnutrition. Future research involving larger populations and extended monitoring is recommended to evaluate the long-term sustainability of these nutritional gains.

## Appendices

**Table 6:** Consolidated assessment tool for maternal sociodemographic, child anthropometry, and IYCF knowledge, attitudes, and practices. IYCF: infant and young child feeding; BM: breast milk; PUC: pre-university college; EBF: exclusive breastfeeding

Part A: Sociodemographic information				
No.	Variables	Options/response space		
1	Name of the child	Open-ended		
2	Gender of the child	(1) Boy/(2) Girl		
3	Date of birth	Open-ended		
4	Age of the child	Open-ended		
5	Phone number	Open-ended		
6	Address	Open-ended		
7	Age of the mother	Open-ended		
8	Caste	(1) General/(2) OBC/(3) SC/ST		
9	Religion	(1) Hindu/(2) Muslim/(3) Christian/(4) Jain/(5) Others		
10	Mother tongue	(1) Kannada/(2) Marathi/(3) Hindi/(4) Konkani/(5) Others		
11	Marital status	(1) Married/(2) unmarried/(3) divorced/(4) widowed		
12	Educational status (mother)	(1) PG/(2) graduate/(3) PUC or diploma/(4) high school/(5) middle school/(6) primary school/(7) illiterate		
13	Occupational status (mother)	(1) Employed/(2) unemployed		
14	Occupation (mother)	(1) Govt./(2) private/(3) self-employed/(4) daily wage/(5) housewife/(6) others		
15	Type of housing	(1) Kaccha/(2) semi-kaccha/(3) pakka/(4) semi-pakka [Hindi]		
16	Family type	(1) Nuclear/(2) joint		
17	Monthly family income	Open-ended		
18	Total family members	Open-ended		
Part B: Anthropometric measurements of children				
1	Height	Open-ended		
2	Weight	Open-ended		
3	Mid-upper arm circumference (MUAC)	Open-ended		
4	Chest circumference	Open-ended		
5	Head circumference	Open-ended		
Part C: Nutritional knowledge of mothers				
1	What should be the first feed of the baby?	(a) Breast milk, (b) honey, (c) sugar water, (d) cow’s milk, and (e) others		
2	What is the right time to initiate breastfeeding?	(a) Within 1 h, (b) within 2-3 h, (c) after 3 days, and (d) I don’t know		
3	What is meant by colostrum?	(a) Thick yellowish fluid, (b) white milk, (c) watery discharge, and (d) no idea		
4	What does colostrum contain?	(a) Proteins, (b) vitamins, (c) fat, (d) essential nutrients and antibodies, and (e) no idea		
5	Exclusive breastfeeding means:	(a) Only breast milk, (b) BM and water, (c) BM and formula, and (d) I don’t know		
6	Exclusive breastfeeding duration:	(a) 2 months, (b) 4 months, (c) 6 months, and (d) 8 months		
7	How long to breastfeed with other foods?	(a) <1 year, (b) 1 year, (c) 1.5 years, (d) 2+ years, and (e) I don’t know		
8	Can breastfeeding prevent disease?	(a) Yes, (b) no, and (c) don’t know		
9	Appropriate time to start complementary foods?	(a) 4th month, (b) before 6 months, (c) after 6 months, and (d) 8th month		
10	Why is complementary food essential?	(a) Satiate hunger, (b) BM inadequate after 6 months, (c) proper growth, (d) all of the above, and (e) don’t know		
11	Should lactating mothers take healthy food?	(a) Yes, (b) no, and (c) don’t know		
12	Is dietary diversity important?	(a) Yes, (b) no, and (c) don’t know		
13	What is micronutrient deficiency?	(a) Insufficient vitamins/minerals, (b) adequate vitamins, and (c) don’t know		
14	Preferred milk other than mother’s milk?	(a) Cow milk, (b) buffalo milk, (c) nothing else, and (d) others		
15	Milk is a rich source of:	(a) Calcium, (b) potassium, (c) protein, (d) all of the above, and (e) don’t know		
16	Green leafy vegetables are rich in:	(a) Calcium, (b) iron, (c) fiber, (d) all of the above, and (e) don’t know		
17	What is anemia?	(a) Low level of Hb, (b) high level of Hb, and (c) I don’t know		
Part D: Attitude toward IYCF				
No.	Question	Agree	Neutral	Disagree
1	BM provides all necessary nutrients for growth.			
2	Breastfeeding should be initiated after one hour of delivery.			
3	Early initiation promotes child development.			
4	Colostrum contains essential nutrients and antibodies.			
5	EBF means only BM without any other food/drink/water.			
6	EBF for the first six months is important.			
7	Breastfeeding can be continued for up to two years.			
8	Start complementary foods after six months.			
9	Lack of diversity increases micronutrient deficiency risk.			
10	Packaged foods/biscuits/chocolates are not nutritious.			
11	Green leafy vegetables improve hemoglobin.			
12	Milk is rich in calcium, protein, and potassium.			
Part E: Feeding practices				
1	Are you breastfeeding your child?	(a) Yes and (b) no		
2	When did you first breastfeed after delivery?	(a) 30-60 mins, (b) 2-24 h, (c) >24 h, and (d) after 3 days		
3	What was the first thing you fed the baby?	(a) Colostrum, (b) honey, (c) water, and (d) others		
4	Did you feed colostrum in the first 3 days?	(a) Yes, (b) no, and (c) don’t know		
5	Have you fed animal colostrum to the baby?	(a) Yes, (b) no, and (c) don’t know		
6	Duration of exclusive breastfeeding practiced:	(a) 6 months, (b) 3 months, (c) 1 month, and (d) 12 months		
7	Total years you will continue breastfeeding:	(a) 1.5 years, (b) 2 years, and (c) >2 years		
8	Do you wash your breasts before feeding?	(a) Yes, (b) no, and (c) don’t know		
9	Feed more frequently when the child is sick?	(a) Yes, (b) no, and (c) don’t know		
10	When did/will you start complementary feeding?	(a) After 1 month, (b) after 6 months, (c) after 10 months, and (d) after 24 months		
11	Frequency of feeding in a day?	(a) Once, (b) twice, and (c) 3+ times		
12	Which milk other than BM?	(a) Cow, (b) buffalo, and (c) don’t know		
13	Frequency of packaged foods/biscuits/sweets?	(a) Every day, (b) 2x/week, (c) once in 15 days, and (d) rarely		
14	Which are the main food groups you give for a 6-23-month-old child			
-	Food group.	Yes		No
-	Breast milk.			
-	Grains, roots, tubers, and plantains.			
-	Pulses (beans, peas, lentils), nuts, and seeds.			
-	Dairy products (milk, formula, yogurt, cheese).			
-	Flesh foods (meat, fish, poultry, organ meats).			
-	Eggs.			
-	Vitamin A-rich fruits and vegetables.			
-	Other fruits and vegetables.			
